# Prevalence of Low Back Pain and Its Related Risk Factors and Disability Following Lumbar Discectomy: A Single-Center Study

**DOI:** 10.7759/cureus.49729

**Published:** 2023-11-30

**Authors:** Fahad A Al Mulhim, Hassan A Alalwan, Abdullah M Alkhars, Adia Almutairi, Mohammed N AlSaeed, Fatimah M Althabit

**Affiliations:** 1 Orthopedic Surgery, King Fahad Hospital Hofuf, Al-Ahsa, SAU; 2 Orthopedic Surgery, King Faisal University, Al-Ahsa, SAU; 3 Orthopedics, King Fahad Hospital Hofuf, Al-Ahsa, SAU; 4 Orthopedics, King Abdulaziz Hospital, Al-Ahsa, SAU; 5 Orthopedics, King Fahad Hospital, Al-Ahsa, SAU

**Keywords:** spine surgery, oswestry low back pain disability index, low back pain disability, discectomy, low back pain

## Abstract

Background

Lumbar disc herniation is considered the most common cause of sciatica, which is a contributing factor to disability. Surgical management of lumbar disc herniation in the form of discectomy is superior to conservative management in terms of better recovery and pain relief. However, recurrence, residual back pain, and disability are common expected complications following surgery. Therefore, this study aims to determine the prevalence of low back pain and its related risk factors and disability following discectomy at King Fahad Hospital in the Al-Ahsa region.

Methodology

A cross-sectional study was conducted at King Fahad Hofuf Hospital in the Al-Ahsa region among patients who were admitted and underwent lumbar spine discectomy in the last six years either due to traumatic or degenerative causes. The study used an anonymous questionnaire consisting of the patient’s sociodemographic data, medical and surgical history, spine disease history, and surgical history. The preoperative Glasgow Coma Scale score, neurological status, and American Spinal Cord Impairment Scale score were noted. In addition, immediate postoperative neurological status and minor complications were recorded. Moreover, more than six months postoperatively, the Numeric Analogue Scale and the Oswestry low back pain disability index questionnaire were administered.

Results

A total of 201 patients were included in the study. The majority of the patients were male (59.7%), with ages ranging from 41 to 60 years (51.7). Most patients underwent one surgery (83.6%) in the form of discectomy alone (90.5%) at L3-L4 (58.7%), for which the intervertebral disc was the most common degenerative indication for surgery. All patients (100%) had low back pain preoperatively, and most patients (50.7%) had no back pain six months postoperatively. Preoperatively, 58.2% had diminished neurological status, while only 29.9% showed a deficit postoperatively. Postoperative low back pain was significantly associated with office-based jobs (p = 0.021, 60.5%) and a high number of surgeries (p = 0.004, 74.1%). The following factors were observed to be risk factors for having lower back pain: six months postoperatively, being unemployed (p = 0.024, odds ratio = 4.38, 338% increased risk), having an office-based job (p = 0.012, odds ratio = 3.98, 298% increased risk), and the underlying cause of the problem being degenerative (p = 0.003, odds ratio = 3.34, 234% increased risk). Low back pain-related severe disability postoperatively was significantly associated with increased age >40 (28-50%; p = 0.045), female gender (p = 0.012, 44.4%), and being unemployed (p = 0.002, 51.4%). The level of disability six months postoperatively was moderate in 40.4% of the patients.

Conclusions

Lumbar discectomy is a successful procedure for relieving low back pain among patients with degenerative spine disease, with an improvement that involves neurological status. However, residual back pain may still occur in less than half of the patients despite appropriate management, such as being unemployed or office-based employees and having multiple spine surgeries. However, low back pain-related disability is often moderate, with increasing severity seen with increased age, being female, and being unemployed.

## Introduction

Low back pain is one of the most common causes of long-term disability worldwide. The most reported cause of low back pain is lumbar disc herniation, which is a degenerative disease caused by the displacement of the disc nucleus pulposus or annulus fibrosis beyond the intervertebral disc space [1]. More than 90% of disc herniation occurs at the L4-L5 or L5-S1 disc space, which compresses the L4, L5, or S1 nerve roots and results in radiculopathy, leading to low back pain that radiates to the posterior leg and dorsal foot, known as sciatica [1,2]. Risk factors for lumbar disc herniation include increasing age, male gender, increased body mass index (BMI), smoking, hard and excessive occupational work, trauma, and abnormal spine posture [3,4]. Disc herniation correlates with aging, as it is considered one of its main causes. The highest prevalence of this disease is among people aged between 30 and 50 years, with a 2:1 male-to-female ratio [5].

Treatment of lumbar disc herniation can be conservative or surgical. However, surgical management may have better recovery and faster rates of pain relief compared to conservative treatment [3,6]. A discectomy for lumbar disc herniation is the most commonly performed spinal surgery. The main principle behind it is to relieve the nerve root compression that is caused by herniation [7]. It is done by removing the offending hernia and repairing the outer annulus of the affected vertebra [7,8]. Lumbar discectomy is considered the most common procedure performed in the United States for patients experiencing sciatica pain. In 2003, 2.1 per 1,000 socialized medicine enrollees received a lumbar discectomy or laminectomy [9].

Despite the good outcome following discectomy, residual back pain and recurrent herniation are the two most common postoperative complications following the surgery [9-11]. Recurrent low back pain following lumbar discectomy might be related to many causes, including recurrent disc herniation involving the operating level, a new disc herniation at other spinal levels, epidural scarring or fibrosis, or other anatomical changes involving the spinal canal [10]. In a previous systemic review, 3%-34% of the patients reported recurrent low back pain within 6-24 months post-discectomy as a short-term outcome, while 5%-36% reported recurrent low back pain two years following discectomy as a long-term outcome. Furthermore, the incidence of recurrent disc herniation at the same level was found in up to 23% of patients, of whom 95% required revision discectomy [9].

Low back pain is usually a distressing symptom that affects the patient’s daily activities and productivity. The effect of pain on the patient’s daily life and work can be expressed as a disability or a reduction in physical functioning [12]. Its effect on an individual’s physical and psychological well-being results in considerable socioeconomic costs [13]. Among patients who underwent discectomy, at three months, none were found to have a significant decline in low back disability compared with baseline. After one year of follow-up, 22% of patients reported clinically important worsening of low back pain disability when compared with the three-month levels [9].

However, previous studies conducted in Saudi Arabia have focused on investigating the prevalence of low back pain, its related causes, and risk factors while understanding the patient’s knowledge of sciatica [14]. Additionally, previous studies were conducted to investigate low back pain-related disability without specifying the leading cause of that pain and without studying the outcome of its management and comparing the pain status pre- and post-management [15]. There is a lack of knowledge locally in terms of investigating the incidence of low back pain and its related risk factors with a clear understanding of its related disability among patients with lumbar disc herniation who underwent discectomy and comparing their pain status pre and postoperatively. This study aims to investigate the prevalence of low back pain, its related risk factors, and disability among patients who underwent lumbar discectomy.

## Materials and methods

Study design, population, and settings

This cross-sectional study was conducted at King Fahad Hofuf Hospital in the Al-Ahsa region, Eastern Province of Saudi Arabia, among patients who underwent spine surgery for lumbar spine discectomy from 2016 to 2021 due to degenerative causes. As no similar previous studies have been conducted at the same center or region, the study utilized a convenient sample size that included all patients who were admitted and underwent lumbar spine discectomy at King Fahad Hofuf Hospital due to degenerative causes of both genders. Patients aged 20 years and above, who were mentally stable, and voluntarily decided to participate were included in the study. However, those who were <20 years old, were managed conservatively, had traumatic causes, had extraspinal causes of back pain or radiculopathy, underwent other spine location surgeries (cervical, thoracic, sacral), passed away, and were not willing to participate were excluded from the study. Hence, the sample size of the study was 395 participants.

Study instrument

This study was conducted according to the ethical considerations and approval of the Deanship of Scientific Research at King Faisal University (reference number: KFU-REC-2022-JUN-ETHICS66). The study was conducted using an anonymous, self-administered questionnaire. The questionnaire was answered by the patients via a phone call, in which the patient’s data were gathered by calling the participants to answer the questions after obtaining informed consent from the patients. The data were collected using Google Forms.

The questionnaire that was used in the study had six sections. The first part included the patient’s sociodemographic data. The second part included the patient’s medical and surgical history. The third part included spinal disease history and surgical history, the preoperative Glasgow Coma Scale, neurological status, and the American Spinal Cord Impairment Scale [16,17]. In the fifth part, immediate postoperative neurological status and minor complications were recorded. Finally, the sixth section contained the Numeric Analogue Scale and the Oswestry low back pain disability index questionnaire to evaluate the disability more than six months postoperatively.

The valid and reliable Oswestry Disability Index (ODI) was scored to assess permanent and functional disability. The Oswestry low back disability questionnaire consists of 10 questions to assess the effect of low back pain on everyday life. Points are combined for a score out of 50. Scores are interpreted as follows: 0-4: no disability, 5-14: mild disability, 15-24: moderate disability, 25-34: severe disability, and 35-50: complete disability. This valid and reliable instrument was used in previous studies globally and locally in Saudi Arabia to evaluate low back pain-related disability [18,19].

Statistical analysis

Data analysis was performed using SPSS version 23 (IBM Corp., Armonk, NY, USA). Frequency and percentages were used to display categorical (qualitative) variables. Minimum, maximum, mean, and standard deviation were used to present numerical (quantitative) variables. An independent t-test and chi-square test were used to test for factors associated with the incidence of lower back pain six months postoperatively and factors associated with lower back pain-induced disability levels according to the Oswestry questionnaire among patients who still have lower back pain postoperatively. Multivariate logistic regression was used to determine the risk factors for still suffering from lower back pain six months postoperatively. The logistic regression model included the following variables: gender, age, occupation, diabetes, osteoporosis, procedure, diagnosis or cause of pain, neurological status preoperatively, and pain rating preoperatively. The level of significance was set at 0.05.

## Results

A total of 201 patients were included in the study. Table 1 shows the sociodemographic profile of the patients. Regarding age, 59 (29.4%) were between 20 and 40 years old, 104 (51.7%) were between 41 and 60 years old, and 38 (18.9%) were 61 years and older. Regarding gender, 120 (59.7%) were males, while 81 (40.3%) were females. Concerning marital status, 21 (10.4%) were single, 165 (82.1%) were married, five (2.5%) were divorced, and 10 (5%) were widowed. Regarding occupation, 66 (32.8%) were unemployed, six (3%) were students, 76 (37.8%) had office-based jobs, 32 (15.9%) had field jobs, and 21 (10.4%) had other jobs. Regarding the level of education, 51 (25.4%) had primary/intermediate school education, 59 (29.4%) had high school education, 72 (35.8%) had bachelor’s degree, three (1.5%) had higher education (Master’s degree/PhD), and 16 (8%) had other educational levels. Concerning the household income, 64 (31.8%) had an income of less than 5,000 SR, 79 (39.3%) had an income between 5,000 and 10,000 SR, 40 (19.9%) had an income between 10,000 and 20,000 SR, and 18 (9%) had an income more than 20,000 SR.

**Table 1:** Sociodemographic profile of the patients (n = 201).

Demographic characteristics	n	%
Age		
20–40 years	59	29.40
41–60 years	104	51.70
61 years and more	38	18.90
Gender		
Male	120	59.70
Female	81	40.30
Marital status		
Single	21	10.40
Married	165	82.10
Divorced	5	2.50
Widowed	10	5.00
Occupation		
Unemployed	66	32.80
Student	6	3.00
Office-based job	76	37.80
Field job	32	15.90
Others	21	10.40
Level of education		
Primary/intermediate school	51	25.40
High school	59	29.40
Bachelor’s degree	72	35.80
Higher education (Master’s/PhD)	3	1.50
Other	16	8.00
Household income		
Less than 1,300 $	64	31.80
1,300–2,600 $	79	39.30
2,600–5,300 $	40	19.90
More than 5,300 $	18	9.00

**Figure 1:**
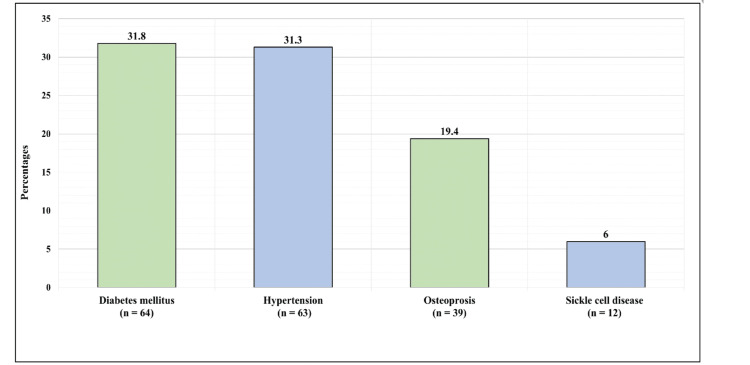
Medical history of the patients.

Figure 1 displays the medical history of the patients. Overall, 64 (31.8%) patients had diabetes mellitus, 63 (31.3%) had hypertension, 39 (19.4%) had osteoporosis, and 12 (6%) had sickle cell disease.

Table 2 presents the spine surgery profile of the patients. Regarding the number of spine surgeries the patients had undergone, 168 (83.6%) had undergone one surgery only, 27 (13.4%) had undergone two surgeries, three (1.5%) had undergone three surgeries, and three (1.5%) had undergone four surgeries. All surgeries were performed in the lumbosacral region of the spine, the exact surgical location of four (2%) patients was between L1 and L2, between L2 and L3 for four (2%), between L3 and L4 for 19 (9.5%), between L4 and L5 for 114 (56.7%), and between L5 and S1 for 60 (29.9%) patients. Overall, 182 (90.5%) patients had undergone discectomy, while 19 (9.5%) patients had undergone discectomy and fixation. The cause of surgery was a traumatic injury for 83 (41.3%) patients, while it was a degenerative issue for 118 (58.7%) patients. As for the mechanism of injury for patients with traumatic injuries, it was road traffic accidents for 29 (34.94%), falls for 28 (33.73%), and carrying heavy objects for 26 (31.33%) patients. Regarding the underlying disease for patients with a degenerative disease, 63 (53.39%) had intervertebral disc herniation, 28 (23.73%) had degenerative spondylolisthesis, 19 (16.1%) had spinal stenosis, four (3.39%) had osteoarthritis, two (1.69%) had pathological fractures, and two (1.69%) had tumors.

**Table 2:** Spine surgery profile (n = 201).

Question	n	%
Q1: Did you undergo any spine surgery?		
Yes	201	100
Q2: How many spine surgeries have you undergone?		
One surgery	168	83.6
Two surgeries	27	13.4
Three surgeries	3	1.5
Four surgeries	3	1.5
Q3: Where was the location of any of the procedures?		
Lumbosacral	201	100
Q4: What was the exact location of the procedure?		
L1–L2	4	2.00
L2–L3	4	2.00
L3–L4	19	9.50
L4–L5	114	56.70
L5–S1	60	29.90
Q5: What was the procedure?		
Discectomy	182	90.5
Both discectomy and fixation	19	9.5
Q6: What was the diagnosis or cause of surgery?		
Traumatic	83	41.3
Degenerative	118	58.7
Q7: If the cause was a traumatic injury, what was the mechanism of injury? (n = 83)		
Road traffic accident	29	34.94
Fall	28	33.73
Carrying heavy object	26	31.33
Q8: If the cause was degenerative, what was the underlying disease?		
Intervertebral disc herniation	63	53.39
Degenerative spondylolisthesis	28	23.73
Spinal stenosis	19	16.10
Osteoarthritis	4	3.39
Pathological fractures	2	1.69
Tumor	2	1.69

**Figure 2:**
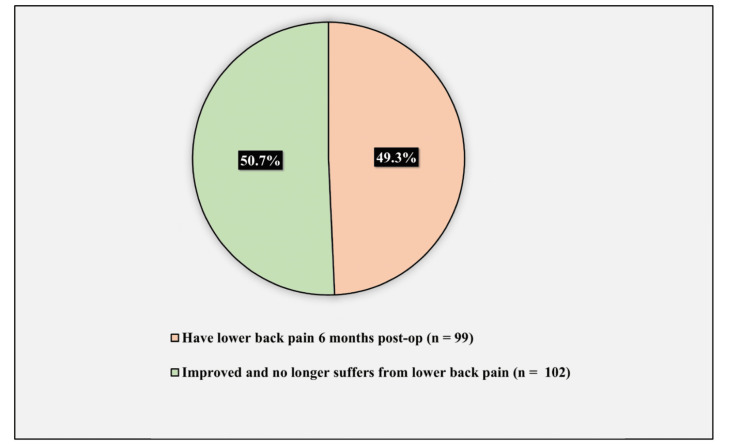
Prevalence of lower back pain six months postoperatively.

Figure 2 demonstrates the prevalence of lower back pain six months postoperatively. The prevalence of lower back pain six months postoperatively was 49.3% (observed in 99 patients), while 50.7% (102 patients) improved and no longer suffered from lower back pain.

Table 3 illustrates the patients’ status preoperatively and postoperatively. Regarding the preoperative status, 201 (100%) patients had lower back pain, and the mean rating of the pain was 7.81 ± 2. Regarding the preoperative neurological status, 69 (34.3%) patients had an intact neurological function, 117 (58.2%) had a diminished neurological function, and 15 (7.5%) had an absent neurological function. Regarding the immediate postoperative status, the general complication incidence rate was 8.5% (affecting 17 patients), while the local (spine) complication incidence rate was 3% (affecting six patients). As for the immediate postoperative neurological status, 138 (68.7%) patients had an intact neurological function, 60 (29.9%) had a diminished neurological function, and three (1.5%) had an absent neurological function. Regarding the six-month postoperative status of patients, the prevalence of lower back pain six months postoperatively was 49.3% (affecting 99 patients), and the mean rating of pain was 5.21 ± 2.08.

**Table 3:** Patients’ status preoperation and postoperation (n = 201).

Question	n	%
Preoperation		
Q1: Did the patient have back pain (preoperative)?		
Yes	201	100
Q2: What was the location of the back pain (preoperative)?		
Lower back pain	201	100
Q3: Pain rating (preoperative) (out of 10)		
Minimum	1	
Maximum	10	
Mean	7.81	
Standard deviation	2.00	
Q4: Neurological status (preoperative)		
Intact	69	34.3
Diminished	117	58.2
Absent	15	7.5
Immediate postoperation		
Q1: Was there any minor complications? (immediate postoperative)		
Yes	17	8.5
No	184	91.5
Q2: Was there any local spine complication? (immediate postoperative)		
Yes	6	3
No	195	97
Q3: Neurological status (immediate postoperative)		
Intact	138	68.7
Diminished	60	29.9
Absent	3	1.5
6 months postoperation		
Q1: Did the patient have back pain (6 months postoperative)?		
Yes	99	49.3
No	102	50.7
Q2: What was the location of the back pain (6 months postoperative)? (n = 99)		
Lower back pain	99	100
Q3: Pain rating (6 months postoperative) (out of 10)		
Minimum	1	
Maximum	10	
Mean	5.21	
Standard deviation	2.08	

**Figure 3:**
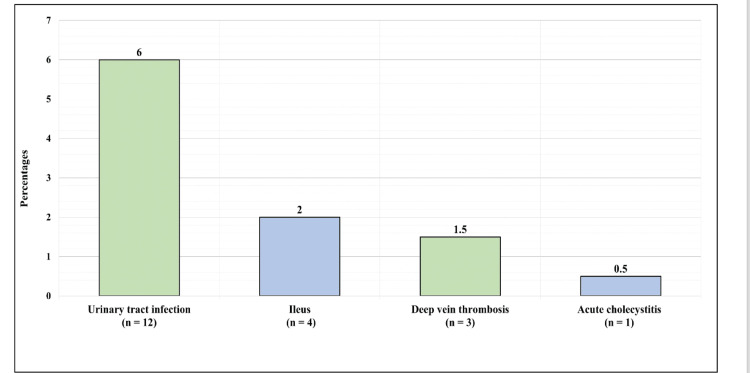
General complications postoperation.

Figure 3 shows the general complication postoperation. Overall, 12 (6%) patients had urinary tract infection, four (2%) had ileus, three (1.4%) had deep vein thrombosis, and one (1.5%) had acute cholecystitis.

**Figure 4:**
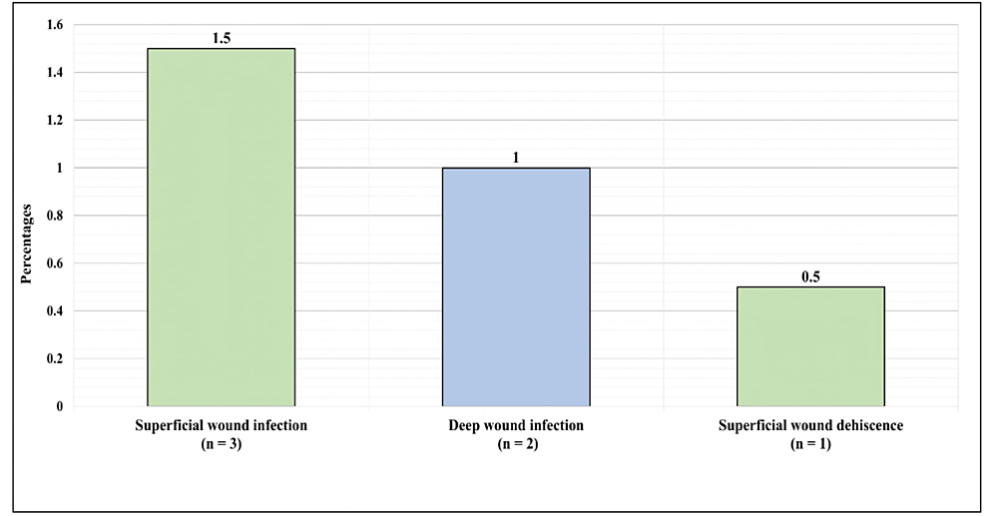
Local complications postoperation.

Figure 4 presents the local complication postoperation. Overall, three (1.5%) had superficial wound infection, two (1%) had deep wound infection, and one (0.5%) had superficial wound dehiscence.

Table 4 displays the low back pain-induced disability assessment using the Oswestry questionnaire (specified for patients who had low back pain postoperatively). The minimum disability score was 0%, the maximum was 78%, and the mean disability score was 34.09% ± 15.67%. As for the disability levels according to Oswestry low back pain disability assessment, 30 (30.3%) had minimal disability (score of 20% and less), 40 (40.4%) had moderate disability (score between 21% and 40%), 21 (21.2%) had severe disability (score between 41% and 60%), and eight (8.1%) were classified as crippled (score between 61% and 80%).

**Table 4:** Low back pain-induced disability assessment using the Oswestry questionnaire specified for patients who had low back pain postoperatively (n = 99).

Question	n	%
Disability score		
Minimum	0	
Maximum	78	
Mean	34.09	
Standard deviation	15.67	
Disability levels according to Oswestry low back pain disability assessment (n = 99)		
Minimal disability	30	30.3
Moderate disability	40	40.4
Severe disability	21	21.2
Crippled	8	8.1

Table 5 compares lower back pain ratings preoperatively and postoperatively among patients who had lower back pain postoperatively. A significant reduction in pain severity was observed among patients who still suffered lower back pain postoperatively (p < 0.001) (preoperative mean severity of pain = 8.1 ± 2 vs. postoperative mean severity of pain = 5.29 ± 2.06).

**Table 5:** Comparison of lower back pain rating preoperatively and postoperatively among patients who had lower back pain postoperatively.

Factor	Pain rating		P-value
	Mean	Standard deviation	
Pain rating (out of 10)			<0.001*
Preoperative	8.1	2.00	
Postoperative	5.29	2.06	
*Significant at level 0.05			

Table 6 illustrates the factors associated with the presence of lower back pain postoperatively. Occupation was significantly associated with the presence of lower back pain six months postoperatively (p = 0.021), where it was observed that those with field jobs had the lowest rate of back pain postoperatively (31.3%), while those with office-based jobs had the highest rate of lower back pain postoperatively (60.5%). The number of spine surgeries the patient underwent was also significantly associated with the prevalence of lower back pain postoperatively (p = 0.004), where it was observed that those with two surgeries had a higher rate of lower back pain compared to those with only one surgery (74.1% vs. 44%). The cause of the problem was also significantly associated with the prevalence of lower back pain postoperatively (p = 0.001), where it was observed that those with a degenerative origin of the problem had a significantly higher rate of lower back pain postoperatively compared to those with traumatic nature of the problem (59.3% vs. 34.9%). Neurological status preoperatively was also significantly associated with the prevalence of lower back pain postoperatively (p < 0.001), where it was observed that those with diminished neurological function had a notably lower rate of lower back pain postoperatively compared to those with intact neurological pain and absent neurological pain. Age, gender, diabetes, osteoporosis, the procedure the patients underwent, and the preoperative pain severity were not significantly associated with the prevalence of lower back pain postoperatively.

**Table 6:** Factors associated with the presence of lower back pain postoperatively.

Factor	Status of lower back pain postoperatively		P-value
	Have back pain	Don’t have back pain	
Age (n, %)			0.891
20–40 years	29 (49.2%)	30 (50.8%)	
41–60 years	50 (48.1%)	54 (51.9%)	
61 years and more	20 (52.6%)	18 (47.4%)	
Gender (n, %)			0.263
Male	63 (52.5%)	57 (47.5%)	
Female	36 (44.4%)	45 (55.6%)	
Occupation (n, %)			0.021*
Unemployed	35 (53%)	31 (47%)	
Office-based job	46 (60.5%)	30 (39.5%)	
Field job	10 (31.3%)	22 (68.8%)	
Having diabetes mellitus (n, %)			0.874
Yes	31 (48.4%)	33 (51.6%)	
No	68 (49.4%)	69 (50.4%)	
Having osteoporosis (n, %)			0.133
Yes	15 (38.5%)	24 (61.5%)	
No	84 (51.9%)	78 (48.1%)	
Number of spine surgeries the patient underwent (n, %)			0.004*
One	74 (44%)	94 (56%)	
Two	20 (74.1%)	7 (25.9%)	
What was the procedure the patient underwent? (n, %)			0.255
Discectomy	92 (50.5%)	90 (49.5%)	
Both discectomy and fixation	7 (36.8%)	12 (63.2%)	
What was the cause of the operation? (n, %)			0.001*
Traumatic	29 (34.9%)	54 (65.1%)	
Degenerative	70 (59.3%)	48 (40.7%)	
Neurological status preoperatively (n, %)			<0.001*
Intact	47 (68.1%)	22 (31.9%)	
Diminished	42 (35.9%)	75 (64.1%)	
Absent	10 (66.7%)	5 (33.3%)	
Preoperative pain rating (mean ± standard deviation)	7.96 ± 0.67	7.65 ± 1.99	0.281
*Significant at level 0.05			

Table 7 shows the factors associated with lower back pain-induced disability levels according to the Oswestry questionnaire among patients who had lower back pain postoperatively. Age was observed to be significantly associated with the disability level (p = 0.045), where it was observed that the older the patients the higher the rate of severe disability or being crippled. Gender was also significantly associated with disability level (p = 0.012), where it was observed that females had a significantly higher rate of severe disability or being crippled compared to males (44.4% vs. 20.6%). The occupation was also significantly associated with disability level (p = 0.002), where it was observed that unemployed patients had the highest rate of severe disability or being crippled (51.4%) compared to patients with office-based jobs (15.2%) and field jobs (20%). Diabetes, osteoporosis, the number of spine surgeries the patients underwent, the procedure the participants underwent, the cause of the operation, preoperative neurological status, and preoperative pain severity were not significantly associated with the level of disability.

**Table 7:** Factors associated with lower back pain-induced disability levels according to the Oswestry questionnaire among patients who had lower back pain postoperatively.

Factor	Disability level		P-value
	Mild to moderate disability	Severe disability or crippled	
Age (n, %)			0.045*
20–40 years	24 (82.8%)	5 (17.2%)	
41–60 years	36 (72%)	14 (28%)	
61 years and more	10 (50%)	10 (50%)	
Gender (n, %)			0.012*
Male	50 (79.4%)	13 (20.6%)	
Female	20 (55.6%)	16 (44.4%)	
Occupation (n, %)			0.002*
Unemployed	17 (48.6%)	18 (51.4%)	
Office-based job	39 (84.8%)	7 (15.2%)	
Field job	8 (80%)	2 (20%)	
Having diabetes mellitus (n, %)			0.361
Yes	20 (64.5%)	11 (35.5%)	
No	50 (73.5%)	18 (26.5%)	
Having osteoporosis (n, %)			0.108
Yes	8 (53.3%)	7 (46.7%)	
No	62 (73.8%)	22 (26.2%)	
Number of spine surgeries the patient underwent (n, %)			0.186
One	52 (70.3%)	22 (29.7%)	
Two	17 (85%)	3 (15%)	
What was the procedure the patient underwent? (n, %)			0.965
Discectomy	65 (70.7%)	27 (29.3%)	
Both discectomy and fixation	5 (71.4%)	2 (28.6%)	
What was the cause of the operation? (n, %)			0.810
Traumatic	21 (72.4%)	8 (27.6%)	
Degenerative	49 (70%)	21 (30%)	
Neurological status preoperatively (n, %)			0.069
Intact	36 (76.6%)	11 (23.4%)	
Diminished	30 (71.4%)	12 (28.6%)	
Absent	4 (40%)	6 (60%)	
Preoperative pain rating (mean + standard deviation)	8.07 ± 1.74	7.69 ± 2.54	0.154
*Significant at level 0.05			

Table 8 displays the multivariate logistic regression (factors predicting the prevalence of back pain postoperatively). The logistic regression model included the following variables: gender, age, occupation, diabetes, osteoporosis, procedure, diagnosis/cause of pain, preoperative neurological status, and preoperative pain rating. The following factors were observed to be risk factors for having lower back pain six months postoperatively: being unemployed (p = 0.024, odds ratio = 4.38, 338% increased risk), having an office-based job (p = 0.012, odds ratio = 3.98, 298% increased risk), and the underlying cause of the problem being degenerative (p = 0.003, odds ratio = 3.34, 234% increased risk). The following factors were observed to be associated with a decreased risk of lower back pain six months postoperatively: suffering from osteoporosis (p = 0.013, odds ratio = 0.25, 75% decreased risk) and a diminished neurological function pre-op (p < 0.001, odds ratio = 0.16, 84% decreased risk).

**Table 8:** Multivariate logistic regression (factors predicting the prevalence of back pain postoperatively).

Factor	P-value	Odds ratio	Confidence interval	
Gender (male vs. female)	0.075	2.27	0.92	5.57
Age (20–40 years is the referent)				
41–60 years	0.443	0.70	0.28	1.74
61 years and more	0.535	1.58	0.38	6.62
Occupation (field job is the referent)				
Unemployed	0.024*	4.38	1.21	15.86
Office-based job	0.012*	3.98	1.36	11.66
Having diabetes mellitus (yes vs. no)	0.425	0.67	0.25	1.79
Having osteoporosis (yes vs. no)	0.013*	0.25	0.08	0.75
Procedure (discectomy vs. both discectomy and fixation)	0.636	0.71	0.17	2.98
Diagnosis/cause (degenerative vs. traumatic)	0.003*	3.34	0.14	0.66
Neurological status preoperatively (intact is the referent)				
Diminished	<0.001*	0.16	0.07	0.37
Absent	0.853	1.16	0.23	5.83
Preoperative pain rating	0.087	1.18	0.98	1.44
* Significant at level 0.05				

## Discussion

The findings of this study conducted at King Fahad Hospital in the Al-Ahsa district of Eastern Province, Saudi Arabia, offer significant insights into the prevalence of low back pain following discectomy as well as the risk factors associated with it. The findings of this study showed that the prevalence of lower back pain six months after surgery was 49.3%, which is in line with the findings of prior studies that have indicated a range of 3% to 36% for recurring back pain after lumbar discectomy [7,15,16]. The study also found work to be a significant risk factor for the occurrence of lower back pain after surgery, with office-based jobs having the highest rate of lower back pain among all occupations (60.5%). Our findings are in line with the findings of prior research, which demonstrated that an increased risk of low back pain is associated with office employment that requires extended periods of sitting with poor ergonomics [17]. Physically demanding work was not found to be a risk factor in the study by Puolakka et al. (2008) [18]. However, self-reported workload, which was dichotomized as either physically light or physically demanding, may not be an accurate and valid measure of the actual workload. It is important to have a better understanding of the physiological and mental demands of a patient’s profession. The combination of this information with data on the patient’s functional ability, such as the individual items that make up the ODI, could further improve the identification of patients who are in danger of losing their capacity to work and assist in the planning of suitable rehabilitation [17].

The number of spinal operations that a patient had was found to have a substantial association with the patient’s experience of lower back pain after surgery, according to the findings of the study. This discovery might be attributable to the fact that having multiple procedures in a short duration might cause greater harm to the tissues and raise the likelihood of problems [18]. In addition, having degeneration as the underlying cause of the problem was a risk factor for experiencing lower back discomfort six months postoperatively. This finding is similar to prior studies [18]. On the other hand, osteoporosis was associated with a decreased risk of lower back pain six months after surgery. This may be because patients who have osteoporosis are more likely to have weaker bones, which results in less pressure being placed on the spine and a reduced risk of lower back pain after surgery [19]. According to the findings of the study, age, gender, and occupation are major characteristics that are connected with a person’s level of disability. In particular, women had a much greater rate of severe handicaps or being crippled when compared to men, which highlights the necessity of tackling socioeconomic variables in the therapy and prevention of low back pain [18]. According to the results of several studies, the incidence of low back pain is more common in women than in men. This result was similar to the study conducted in the El-Dakahlia governorate, where the prevalence of low back pain was higher among female patients (62.8%) than among male patients. Alemam (2022) found that low back pain was significantly more common in females than in males. The percentage of females diagnosed with low back pain was 73.4%, while the percentage of males diagnosed with low back pain was 47.8% [20].

According to the findings of the study by Sikiru and Hanifa (2010), the incidence of low back pain was more common in females (68%) [21]. The hormonal shifts, gynecological difficulties, and childbirth that women go through might be able to help explain why there is a higher prevalence of low back pain in females [21]. According to Heuch et al. (2013), these factors might help explain why women have a higher risk of lower back pain than men [22]. In addition, there is a possibility that socioeconomic factors, such as occupation, job expectations, and stress associated with work, may contribute to the higher prevalence of low back pain in females. Women, for instance, are more likely to work in jobs that involve performing repetitive tasks or standing for long periods, both of which may raise the risk of lower back pain [22]. According to Puolakka et al. (2008), the higher frequency of severe disability or being crippled among females compared to males shows the need to address socioeconomic issues in the management and prevention of low back pain [18]. Specifically, interventions that aim to reduce job demands and stress related to work, as well as those that promote physical activity and healthy lifestyle behaviors, may be particularly beneficial for women who suffer from low back pain.

Older age was a risk factor for back-related retirement. This may be because of the decline in performance capability that comes with advancing age as well as the increased possibility of incapacitating ailments and diseases [18,23]. The link between getting older and developing lower back pain is complicated and can be further complicated by several other factors. According to the findings of the study by Alemam (2022), the prevalence of LBP was higher in older people when compared to younger age groups [20]. These findings are in line with those reported by Heissam (2015) and Emmanuel and Ezhilarasu (2016) [24,25]. On the other hand, Lodato and Kaplan (2013) found that the prevalence of low back pain was highest in the third decade, increased with age, reaching a peak in the age group of 60-65 years, and then underwent a progressive reduction after that [26]. On the other hand, several studies have found that the risk of experiencing low back pain is highest between the ages of 20 and 40 years old [27,28]. Differences in the research populations, study designs, and techniques of data analysis could be responsible for the apparent inconsistencies in the conclusions of these studies. Physical issues associated with aging may be one possible reason for the relationship between older age and lower back pain. These age-related physical issues may have a substantial impact on the development of low back pain. According to Puolakka et al. (2008), performance capacity decreases with age, and the long life of older individuals is often accompanied by debilitating disorders and diseases, which may increase the risk of developing low back pain [18]. Furthermore, Puolakka et al. (2008) found that older individuals were more likely to have a family history of low back pain. In addition, elderly patients have a lower likelihood of benefiting from vocational rehabilitation because, on average, they have a lower level of education and are less motivated to make a change in their employment situation than younger people [18]. Employers may also be reluctant to hire older individuals, which may add to the increased likelihood of those individuals becoming eligible for a disability pension as a result of low back pain. The greater prevalence of low back pain among those who are older may be connected to the cumulative effects of physical wear and tear on the spine over time. This can include age-related changes in the spinal discs, bones, and joints, which can lead to degenerative conditions such as osteoarthritis and spinal stenosis. These changes may result in nerve compression and inflammation, both of which may contribute to the development of low back pain. However, lower back pain can be correlated to many other conditions or disorders other than herniated intervertebral discs, such as cauda equina and spinal stenosis [29].

The outcomes of this study highlight the importance of undergoing appropriate rehabilitation and preventative measures after having a discectomy to address the risk factors related to low back pain. It has been demonstrated that specific exercise therapy of the back muscles can significantly reduce pain and disability after disc surgery [30,31]. Additionally, an early return to vigorous activities is possible in the majority of patients without increasing the rate of complications [31]. Nevertheless, the findings of the study suggest that the standard sick leave of two months that was mandated during one of the studies may be excessively long in many situations [31]. For this reason, a more individualized approach to the planning of rehabilitation that takes into consideration the patient’s particular risk factors and the requirements of their employment is required to prevent the patient’s loss of work capacity [17].

Limitations

A significant limitation of this study is its cross-sectional design, which precludes establishing a causal association between the risk factors and the incidence of low back pain or disability following discectomy. The use of self-reported data, which may be susceptible to recall bias or social desirability bias, is another potential restriction. In addition, the study’s generalizability may be limited because it only included patients who received lumbar spine discectomy at King Fahad Hofuf Hospital in the Al-Ahsa region and excluded patients who were treated conservatively or who underwent other spine location procedures. All patients who underwent lumbar spine discectomy within the past six years were used as a convenient sample, which may not be typical of the general population. Lastly, the study did not evaluate some potential risk factors for LBP and disability, such as physical activity level, smoking status, and BMI, which may limit the ability to draw conclusions regarding the overall risk factors for low back pain and disability following discectomy.

## Conclusions

This study sought to assess the incidence of low back pain and its associated risk factors and disability after discectomy at King Fahad Hospital in the Al-Ahsa district. The study found that the majority of patients were males between the ages of 41 and 60 and that the majority received a single discectomy procedure. The study also revealed that all patients experienced low back pain before surgery and that the majority had no back pain six months after surgery. Unemployment, office-based employment, and a degenerative underlying cause of the disease were identified as risk factors for developing low back pain six months after surgery. Low back pain and severe disability following surgery were substantially related to age >40 years, female gender, and unemployment. Most patients had a moderate degree of impairment six months after surgery.
